# Brief Review on the Connection between the Micro-Canonical Ensemble and the *S_q_*-Canonical Probability Distribution

**DOI:** 10.3390/e25040591

**Published:** 2023-03-30

**Authors:** Angel R. Plastino, Angelo Plastino

**Affiliations:** 1 CeBio y Departamento de Ciencias Básicas, Universidad Nacional del Noroeste de la Província de Buenos Aires, UNNOBA, CONICET, Roque Saenz Peña 456, Junin 6000, Argentina; 2 Facultad de Ciencias Exactas, Departamento de Física, UNLP and CONICET-CCT-IFLP, La Plata 1900, Argentina

**Keywords:** generalized entropies, micro-canonical ensemble, *S_q_* non-additive entropies

## Abstract

Non-standard thermostatistical formalisms derived from generalizations of the Boltzmann–Gibbs entropy have attracted considerable attention recently. Among the various proposals, the one that has been most intensively studied, and most successfully applied to concrete problems in physics and other areas, is the one associated with the $S_{q}$ non-additive entropies. The $S_{q}$-based thermostatistics exhibits a number of peculiar features that distinguish it from other generalizations of the Boltzmann–Gibbs theory. In particular, there is a close connection between the $S_{q}$-canonical distributions and the micro-canonical ensemble. The connection, first pointed out in 1994, has been subsequently explored by several researchers, who elaborated this facet of the $S_{q}$-thermo-statistics in a number of interesting directions. In the present work, we provide a brief review of some highlights within this line of inquiry, focusing on micro-canonical scenarios leading to $S_{q}$-canonical distributions. We consider works on the micro-canonical ensemble, including historical ones, where the $S_{q}$-canonical distributions, although present, were not identified as such, and also more resent works by researchers who explicitly investigated the $S_{q}$-micro-canonical connection.

## 1. Introduction

To speak about things, it is often useful to have names for them. Otherwise, one faces a plight similar to that of Garcia Marquez’s celebrated Macondo characters, who lived when “*the world was so recent that many things lacked names, and in order to indicate them it was necessary to point*” [1]. One of the tasks of scientists is to identify and baptize things, including abstract mathematical objects, that deserve to have a name. The probability distributions, nowadays called *q*-exponentials and *q*-Gaussians (*q*-distributions, for short), constitute nice illustrations of physically relevant mathematical objects that, for a long time, lacked a deserved name. Since the very inception of statistical mechanics, the *q*-distributions are discernible in the scientific literature. These distributions are closely related to the micro-canonical ensemble, and are central to some standard derivations of the Gibbs canonical probability distribution from the micro-canonical one. The relevance of the *q*-distributions, however, remained unrecognized for more than a century. The unnoticed distributions remained unnamed.

In 1988 Tsallis advanced a thermo-statistical formalism based on the non-additive entropy $S_{q}$, characterized by the parameter *q* [2]. The new formalism soon attracted the attention of a few adventurous theoreticians because of its mathematical elegance and its physically appealing features. Then, after some concrete applications were identified in the early and mid 1990s, research activity on the $S_{q}$-thermo-statistics increased dramatically, and the field expanded in myriad new directions [3]. In particular, the connection with the micro-canonical ensemble describing finite systems, discovered in 1994 [4], established a direct link between Tsallis theory and some basic ideas of statistical mechanics dating from the very beginnings of this branch of physics. It became clear that the probability distributions (or densities) optimizing the $S_{q}$ entropy were already present in some early works by the founding fathers of statistical mechanics, as well as in some modern textbooks. Nowadays, those distributions, first identified as important in their own right by Tsallis in 1988, are endowed with well-deserved short, catchy names: *q*-exponentials and *q*-Gaussians (the latter are special instances of the former).

The link between the micro-canonical ensemble and either the *q*-exponential or *q*-Gaussians has been, since 1994, investigated by several researchers, from diverse points of view. The aim of the present effort is to provide a brief review of this subject, summarizing its historical background, and discussing recent developments.

This paper is organized in the following way. In Section 2, we describe briefly the $S_{q}$-thermo-statistical formalism and the probability distributions that optimize the $S_{q}$ entropy. The basic connection between the $S_{q}$-thermostatistics and the micro-canonical ensemble is reviewed and explained in Section 3. In Section 4, we provide some historical background, on works prior to 1994, where the *q*-exponential related to the micro-canonical ensemble can be identified. Recent developments on these matters are reviewed in Section 5. Finally, some concluding remarks are given in Section 6.

## 2. Probability Distributions Optimizing the $S_{q}$ Non-Additive Entropies

Generalizations of the maximum entropy principle based on non-standard entropies [5,6,7,8,9,10,11] have been applied to the study of a variety of systems and processes in physics and other fields, especially in relation to complex systems [3,12,13]. This line of endeavor started in earnest around the mid and late 1990s, stimulated, to a large extent, by the early applications of a generalized thermostatistics advanced by Tsallis in 1988. Within the Tsallis proposal, the canonical probability distributions arise from the optimization of the $S_{q}$ non-additive entropies [2]. The $S_{q}$-based thermostatistics has been applied successfully to diverse fields, including physics, astronomy, and biology, among various others [14,15,16,17,18,19]. Of all the thermo-statistics derived from generalized entropic forms, the thermo-statistics associated with the $S_{q}$ entropies has been the one that attracted more attention and the one that generated the largest and most diverse set of fruitful applications. In this section, we shall briefly review the main features of the $S_{q}$-thermo-statistics.

The $S_{q}$-thermo-statistics is derived from the non-additive, entropy $S_{q}$ [3] which is given by the expression

(1) $$S_{q}\;=\;\frac{k}{q-1}\sum_{i=1}^{W}\left(p_{i}-p_{i}^{q}\right),$$

where the parameter *q* determines the degree of non-additivity exhibited by the entropy, the constant *k* determines both the dimensions and the units in which the entropy is measured, and $\left\{p_{i},\;\;i=1,\ldots ,W\right\}$ constitutes a set of normalized probabilities associated with a system that has W microstates. Here, we assume that k=1. In the limit q→1, the standard Boltzmann–Gibbs entropy is recovered: $S_{1}=-k\sum_{i=1}^{W}p_{i}\ln p_{i}=S_{\mathrm{BG}}$. Central to the $S_{q}$ thermo-statistical formalism are the *q*-logarithm and the *q*-exponential functions. The *q*-logarithm is defined as

(2) $$\ln_{q}\left(x\right)\;=\;\frac{1-x^{1-q}}{q-1}\;,$$

and its inverse function, the *q*-exponential, is given by

(3) $$\exp_{q}\left(x\right)={\left[1+\left(1-q\right)x\right]}_{+}^{\frac{1}{1-q}},$$

where

(4) $${\left[1+\left(1-q\right)x\right]}_{+}^{\frac{1}{1-q}}=\left\{\begin{array}{cc} {\left[1+\left(1-q\right)x\right]}^{\frac{1}{1-q}}\;, & \mathrm{if}\;1+(1-q)x>0\;,\\ 0\;, & \mathrm{if}\;1+(1-q)x\leq 0\;. \end{array}\right.$$

The *q*-logarithm and the *q*-exponential functions are inextricably linked to the constrained optimization of the $S_{q}$ entropies [3]. It is worth mentioning that the $S_{q}$ entropy itself can be expressed in terms of *q*-logarithms,

(5) $$S_{q}\;=\;k\sum_{i=1}^{W}p_{i}\ln_{q}\left(1/p_{i}\right)\;=\;k\;\langle \ln_{q}\left(1/p_{i}\right)\rangle .$$

In the limit q→1, the above expression coincides with the well-known one, $S_{\mathrm{BG}}=k\sum_{i=1}^{W}p_{i}\ln\left(1/p_{i}\right)$.

The $S_{q}$ thermo-statistics revolves around the optimization of the entropic form $S_{q}$ under appropriate constraints. The concomitant variational problem can be formulated in terms of standard linear constraints, or in terms of power-law nonlinear constraints related to escort distributions [3]. For our present purposes, which are to consider some connections between the $S_{q}$ formalism and the micro-canonical ensemble, it is convenient to consider standard linear constraints. The use of linear constraints also makes it easier to consider more general entropic functionals, for which it is still not well understood what type of escort mean values should be used. $S_{q}$-based canonical probability distribution is obtained when the $S_{q}$ entropy is optimized under the constraints imposed by normalization,

(6) $$\sum_{i=1}^{W}\;p_{i}\;=\;1,$$

and by the system’s mean energy. If the *i*th microstate of the system has energy $\epsilon_{i}$ and probability $p_{i}$, the mean energy is given by

(7) $$E\;=\;\sum_{i=1}^{W}\;p_{i}\;\epsilon_{i}.$$

We assume that the energies of the system are bounded from below, and that for all states, $\epsilon_{i}\geq 0$. The optimization of the entropy $S_{q}$ under the constraints (6) and (7) leads to

(8) $$\delta \left[S_{q}\;-\;\alpha \left(\sum_{i=1}^{W}\;p_{i}\right)-\beta E\right]\;=\;0,$$

where α and β are the Lagrange multipliers associated with the constraints of normalization (6) and the mean energy (7). It follows from (8) that

(9) $$p_{i}^{q-1}=\frac{1}{q}\;\left[1\;-\;\left(q-1\right)\left(\alpha +\beta \epsilon_{i}\right)\right],$$

and

(10) $$p_{i}={\left(\frac{1}{q}\right)}^{\frac{1}{q-1}}\;{\left[1\;-\;\left(q-1\right)\left(\alpha +\beta \epsilon_{i}\right)\right]}_{+}^{\frac{1}{q-1}},$$

which constitutes the $S_{q}$-canonical probability distribution. The probabilities (10) can be recast as

(11) $$p_{i}=C\;{\left[1\;-\;\left(q-1\right)\tilde{\beta }\epsilon_{i})\right]}_{+}^{\frac{1}{q-1}},$$

where $C={\left[\left(\frac{1}{q}\right)\frac{\beta }{1\;-\;(q-1)\alpha }\right]}^{\frac{1}{q-1}}$ and $\tilde{\beta }=\frac{\beta }{1\;-\;(q-1)\alpha }$. Expression (11) for the $S_{q}$-canonical probabilities coincides with the one obtained by Tsallis in his seminal paper of 1988 [2] (compare (11) with Equation (12) from [2], making the identifications $\tilde{\beta }\to \beta$ and $C\to 1/Z_{q}$). In what follows, we shall use expression (11), focusing on scenarios satisfying q>1 and $\tilde{\beta }>0$, which, as we shall see, are related to the micro-canonical ensemble. It is also worth emphasizing that the probabilities (11) can be expressed in the standard *q*-exponential form, as

(12) $$p_{i}=C\;\exp_{\tilde{q}}\left[-\tilde{\beta }\epsilon_{i}\right],$$

where

(13) $$\tilde{q}\;=\;2-q.$$

That is, the expression (11) constitutes just a particular way of parameterizing a *q*-exponential probability distribution. Since, as we already explained, we are going to use the parameterization (11), all the *q*-values mentioned in this review correspond to this parameterization. In order to compare our *q*-values with those of the authors, who use the parameterization (12), one has to apply the relation (13). In what follows, when we use (11) or (12), we shall drop the tilde from $\tilde{\beta }$. When deriving (11), we considered a set of discrete energy levels. In the case of classical Hamiltonian systems with a Hamiltonian *H* and continuous phase space, the optimization of the $S_{q}$ entropy under the normalization and mean energy constraints leads to a phase space $S_{q}$-canonical probability density of the form

(14) $$F\left(\omega \right)=C\;{\left[1\;-\;(q-1)\beta H(\omega )\right]}_{+}^{\frac{1}{q-1}},$$

where $\omega =(q_{i},q_{2},\ldots ;p_{1},p_{2},\ldots )$ denotes the set of generalized coordinates and conjugate momenta.

## 3. The Micro-Canonical Path towards the $S_{q}$-Canonical Distribution

Now, we shall review how the $S_{q}$ canonical distribution can arise from the micro-canonical ensemble. We shall consider a composite system A+B consisting of two weakly coupled subsystems *A* and *B*. The subsystem *A* has energy levels $\epsilon_{i},\;\;\;i=1,2,\ldots$. The “total” system A+B is described by the Gibbs micro-canonical ensemble, and has a total energy lying in the interval $\left(E_{T}-\Delta ,E_{T}+\Delta \right)$ with $\Delta <<E_{T}$. The level distribution of subsystem B is assumed to be quasi-continuous. Under these assumptions, it is possible to show that, if the number of states of system *B* having energies less or equal to *E* grows as a power of *E*, then the marginal probability distribution associated with subsystem *A* has the same form as the $S_{q}$-canonical distribution (11) (or, equivalently, as (12)).

The proof of the above statement is based on the fact that the probability $p_{i}$ of finding the system *A* in a particular state *i* with energy *i* is proportional to the total number ν of configurations of the complete system A+B that are compatible with the state of affairs. In order to determine ν, we introduce the assumption, already mentioned, that the number N(E) of states of system *B* having energies less or equal to *E* complies with the power law,

(15) $$N\left(E\right)\;\propto \;E^{\eta },$$

The above power law implies that the number of states of system *B* having energies within the range (E−Δ,E+Δ) is proportional to

(16) $$2\Delta E^{\eta -1},$$

from which it follows that the number ν of configurations of the total system A+B compatible with finding *A* in a state with energy $\epsilon_{i}$ satisfies,

(17) $$\nu \;\propto \;{\left(E_{T}-\epsilon_{i}\right)}^{\eta -1}.$$

It is plain from the above relation that the probabilities $p_{i}$ to find the system *A* is in its different states i=1,2,…, comply with

(18) $$\frac{p_{i}}{p_{j}}\;=\;\frac{{\left(E_{T}-\epsilon_{i}\right)}^{\eta -1}}{{\left(E_{T}-\epsilon_{j}\right)}^{\eta -1}}\;=\;\frac{{\left[1-\left(\epsilon_{i}/E_{T}\right)\right]}^{\eta -1}}{{\left[1-\left(\epsilon_{i}/E_{T}\right)\right]}^{\eta -1}}.$$

After introducing a normalization factor C, defined by the relation

(19) $$C^{-1}\;=\;\sum_{i}\;{\left[1-\left(\epsilon_{i}/E_{T}\right)\right]}^{\eta -1},$$

the probabilities $p_{i}$, associated with the states of system *A*, can be finally cast as

(20) $$p_{i}\;=\;C\;{\left[1-\left(\epsilon_{i}/E_{T}\right)\right]}^{\eta -1}.$$

Setting now

(21) $$q\;=\;\frac{\eta }{\eta -1},$$

and

(22) $$\beta \;=\;\frac{\eta -1}{E_{T}},$$

it follows that the marginal probability distribution describing the system *A* has the same form as the $S_{q}$-canonical distribution, given by (11) (or, alternatively, by (12)).

The main assumptions made in the above arguments can be summarized as follows:

- We have two weakly interacting systems, *A* and *B*, jointly described by the micro-canonical ensemble.
- The energy level distribution of subsystem B is quasi-continuous, and the number of states of system *B* with energy less than or equal to *E* grows as a power $E^{\eta }$.

Some comments are in order with regards to the second assumption. First, systems complying with that assumption are not rare. Systems such that the number of states grows with a power η of the system’s energy can be realized in a number of ways. Particular examples are the following: a system *B* consisting of *N* quantum harmonic oscillators, for which one has η=N; a set of *N* free non-relativistic quantum particles moving in a *D*-dimensional box, for which η=DN/2; and a system of *N* rigid, quantum plane rotators, for which η=N/2. As can be appreciated in these examples, in order to exhibit the desired behavior, *B* has to be finite. If one interprets *B* as a heat bath, it follows that one of the physical scenarios leading to the $S_{q}$-canonical distribution is the one corresponding to systems in equilibrium with a finite heath bath. This is, indeed, a sensible interpretation. The finite-bath interpretation is not, however, essential for our present purposes, and we shall not emphasize it. In order to establish the connection between the $S_{q}$-canonical distribution and the micro-canonical ensemble, it is enough to consider a system consisting of two weakly coupled subsystems, in which one of the subsystems (our system *B*) is such that the number of states grows as a power of energy.

Notice that in all the above-mentioned instances of a system *B* for which the number of states depends on *E* according to the appropriate power-law behavior, the exponent η is proportional to the number *N* of constituents of *B*. If one lets N→∞ and $E_{T}\to \infty$, keeping the quotient $E_{T}/N$ constant, one also has η→∞, with $\eta /E_{T}$ constant. It can be verified that, if one takes that limit, then *q* goes to unity, and the probability distribution associated with system *A* becomes the standard canonical exponential one,

(23) $$p_{i}\;=\;\frac{1}{Z}\;\exp\left(-\beta \epsilon_{i}\right),$$

where

(24) $$Z\;=\;\sum_{i}\exp\left(-\beta \epsilon_{i}\right).$$

If *B* is interpreted as an infinite heath bath (resulting from taking the thermodynamic limit N→∞ and $E_{T}\to \infty$), the above argument is, essentially, the one provided in many textbooks in order to derive the Gibbs canonical distribution (23). In particular, this is the case in Feynman’s celebrated lectures on statistical mechanics [20] (for instance, *q*-distributions are clearly identifiable in page 4 of [20], although they are not explicitly parameterized, nor referred to, as “*q*-distributions”, nor are they, for that matter, given any specific name). The *q*-distributions appear and play a central role in these derivations, although this fact usually remains unnoticed because the connection with the $S_{q}$-based thermostatistics is not recognized. Since 1994, the connection between the $S_{q}$-thermo-statistics and the micro-canonical ensemble has been investigated by several researchers. Baranger, an outstanding contributor to $S_{q}$-thermo-statistics, and an influential commentator on the field [3], hailed the $S_{q}$-canonical-micro-canonical connection as a key ingredient in answering the question “*Why Tsallis Statistics?*”. In a paper published in 2002, Baranger re-visited the argument advanced in [4] and made the bold conjecture that the argument may be extended beyond equilibrium scenarios, helping to explain the phenomenological success of Tsallis statistics in describing diverse non-equilibrium situations [21]. Baranger suggested that, during an out-of-equilibrium process, a subsystem may interact with a finite number of effective degrees of freedom of the rest of the system, establishing a quasi-equilibrium situation described by the $S_{q}$-statistics.

Our previous arguments were formulated in terms of systems having discrete energy levels. The main arguments, however, are still valid if one considers classical Hamiltonian systems with a continuous phase space. Let us consider a classical composite system A+B, consisting of two weakly interacting subsystems, governed by a Hamiltonian $H=H_{A}\left(\omega_{A}\right)+H_{B}\left(\omega_{B}\right)$, where $\omega_{A}$ denotes the set of canonical phase-space variables describing the state of system *A*, and $\omega_{B}$ stands for the set of variables describing the state of system *B*. Let us assume that the volume $\Phi_{B}\left(E\right)$ in the phase space of system *B*, corresponding to $H_{B}\left(\omega_{B}\right)\leq E$, is proportional to a power η of *E*. That is,

(25) $$\Phi_{B}\left(E\right)\;=\;\;\int_{H(\omega_{B})\leq E}\;\;\;d\omega_{B}\;\propto \;E^{\eta },$$

where $d\omega_{B}$ denotes the volume element in the phase space of system *B*. If the condition (25) is satisfied, one can follow essentially the same argument as before, and reach a similar conclusion: if the composite system A+B is described by the micro-canonical ensemble with total energy $E_{T}$, then the marginal probability density corresponding to system *A* has the $S_{q}$-canonical form

(26) $$F_{A}\left(\omega_{A}\right)=C\;{\left[1\;-\;\left(q-1\right)\beta H_{A}\left(\omega_{A}\right)\right]}_{+}^{\frac{1}{q-1}},$$

where C is an appropriate normalization constant, and *q* and β are related to η and $E_{T}$ through Equations (21) and (22). Note that the above setting is applicable even in situations in which *A* and *B* do not, strictly speaking, represent subsystems of a Hamiltonian system. The above results are also applicable when *A* denotes a subset of the canonical variables characterizing the system, *B* denotes the remaining canonical variables, and the total Hamiltonian can be separated as $H=H_{A}+H_{B}$, with the term $H_{A}$ depending only on the variables associated with *A*, the term $H_{B}$ only on the variables associated with *B*. Then, if one assumes that the total system is in the micro-canonical ensemble, and that the volume $\Phi_{B}\left(E\right)$ of the region determined by $H_{B}\leq E$ (the volume $\Phi_{B}\left(E\right)$ is computed with respect to the set of canonical variables associated with *B*) grows as a power η of *E*, the marginal probability distribution for the canonical variables in *A* has the $S_{q}$-canonical shape (26). As an example, we can consider a classical Hamiltonian system in which the kinetic energy is a homogeneous quadratic function of the momenta, and does depend on the configuration coordinates. If *A* denotes the *n* configuration coordinates of the system, and *B* denotes the set of *n* momenta, then one has that $\Phi_{B}\left(E\right)\propto E^{n/2}$. Consequently, if the system is described by the micro-canonical ensemble, the marginal probability density for the configuration coordinates is proportional to ${\left[1-\left(V/E_{T}\right)\right]}^{\frac{n}{2}-1}$, where *V* is the system’s potential energy, and $E_{T}$ is the total energy (here, *V* plays the role of $H_{A}$). We see that the configuration probability density is a *q*-exponential with q=n/(n−2). As we shall see in the next section, this scenario was already discussed by Maxwell in 1879, although the connection with the $S_{q}$-statistics was not recognized at the time.

A paradigmatic scenario (closely related to the previous example) leading to the $S_{q}$-canonical distribution (26) is given by a composite classical Hamiltonian system A+B, where the subsystem *B* consists of *N* free particles of mass *m* moving in a *D*-dimensional box (that is, *B* is a *D*-dimensional finite classical ideal gas). In such a case, one can see that the volume $\Phi_{B}\left(E\right)$ given by (26), is proportional to the volume of a ND-dimensional hyper-sphere of radius $E^{1/2}$. That is,

(27) $$\Phi_{B}\left(E\right)\;\propto \;E^{ND/2}.$$

Therefore, the power η is equal to ND/2. It then follows that, if the composite A+B is jointly described by the micro-canonical ensemble with total energy $E_{T}$, the probability density $F_{A}\left(\omega_{A}\right)$ associated with subsystem *A* is proportional to

(28) $${\left[1-\frac{H_{A}}{E_{T}}\right]}^{\eta -1}\;=\;{\left[1-\frac{H_{A}}{E_{T}}\right]}^{\frac{ND-2}{2}}.$$

The density $F_{A}\left(\omega_{A}\right)$ is thus of the $S_{q}$-canonical form (26), with the *q*-index given by (21), yielding,

(29) $$q\;=\;\frac{\eta }{\eta -1}\;=\;\frac{ND}{ND-2}$$

As already mentioned, the early successes of the $S_{q}$ theory motivated the exploration of alternative thermostatistical frames based on other entropic forms, and also the comparative investigation of the structural features exhibited by general entropic variational principles. The aim of these latter endeavors was to clarify which are the properties that are shared by large families of entropies, or even that are universal, and, on the other hand, which are the distinguishing properties that characterize particular entropies, such as the $S_{q}$ ones. Diverse aspects of entropy optimization principles were investigated in this regard. See, for instance [22,23] and references therein. Within this context, it is natural to ask whether the connection with the micro-canonical ensemble is a specific feature of the $S_{q}$-thermostatistics or, on the contrary, is a feature shared by larger families of thermo-statistical formalisms. It is clear, on the one hand, that the connection with the $S_{q}$-thermostatistics arises from the power law behavior of the function N(E) (Equation (15)) describing the number of states of system *B* with energies less or equal *E*. If the function N(E) associated with *B* does not exhibit a power-law behavior, the probability distribution describing the system *A* will not be $S_{q}$-canonical. It will have a different form, which may be interpreted as optimizing an entropic measure different from $S_{q}$. In that sense, the link with the micro-canonical ensemble may be shared by other entropies. On the other hand, systems whose energy levels (or phase-space volumes) do exhibit the appropriate power-law behavior, and consequently lead to $S_{q}$-canonical distributions, are not rare in nature. As we shall see in the following sections, scenarios in which the $S_{q}$-canonical distribution arises from the micro-canonical ensemble have been discovered and investigated by many researchers, including some of the pioneers of statistical mechanics. This situation, not shared by other generalized entropies, suggests that there may be, perhaps, something unique to the link between the $S_{q}$-thermostatistics and the micro-canonical ensemble.

## 4. When *q*-Exponentials Lacked a Name: From Maxwell to the Mid 1990s

Interesting examples of *q*-exponential distributions can already be identified in one of Maxwell’s pioneering works on statistical mechanics, his celebrated paper “On Boltzmann’s Theorem on the Average Distribution of Energy in a System of Material Points”, published in 1879 [24] (the paper can also be found in Maxwell’s collected scientific works [25]). In this paper, Maxwell developed a statistical treatment for the dynamics of a closed system of interacting particles having a constant total energy. Maxwell considered a large number of copies of the system, distributed uniformly on a phase-space hyper-surface of constant energy. In essence, Maxwell’s idea was to describe the system by recourse to what, in modern parlance, we now call the micro-canonical ensemble. In Equation (41) of [24], Maxwell determined that “the number of systems whose configuration is specified by the variables $b_{1},\ldots ,b_{n}$ while the momenta may have any values consistent with the equation of energy” (in Maxwell’s notation $b_{1},\ldots ,b_{n}$ denotes the complete set of configuration coordinates) [24]. In other words, Equation (41) of Maxwell’s paper corresponds to the marginal distribution in configuration space, obtained from the micro-canonical ensemble after tracing over the particles’ momenta. Maxwell proved that the distribution in configuration space is proportional to

(30) $${\left(E-V\right)}^{\frac{n-2}{2}},$$

where *E* is the system’s total energy, and $V(b_{1},\ldots ,b_{n})$ is the the system’s total potential energy. If the potential energy is bounded from below, we can choose the zero of energy in such a way that E,V>0. The configuration density $F(b_{1},\ldots ,b_{n})$ can then be written as

(31) $$F\left(b_{1},\ldots ,b_{n}\right)=C{\left(1-\frac{V(b_{1},\ldots ,b_{n})}{E}\right)}^{\frac{n-2}{2}},$$

which is clearly a *q*-exponential of the potential energy *V*, with a *q* index given by

(32) $$q\;=\;\frac{n}{n-2}.$$

It goes without saying that Maxwell himself, working a century before Tsallis, did not identify the configuration density as a *q*-exponential.

As already emphasized, *q*-distributions play an important role in many textbooks’ derivation of the Gibbs canonical distribution [20]. They can also be found in some remarkable works from the 1990s on the micro-canonical approach to finite classical Hamiltonian systems [26,27], although in none of these works were the *q*-distributions identified as such, nor was their connection with the $S_{q}$-thermo-statistics discussed.

In [26], the authors investigate the micro-canonical approach to classical systems consisting of a small number of particles. The authors point out that the momentum distribution of small systems described by the micro-canonical ensemble is non-Maxwellian. The authors prove that the single-particle momentum distribution (see Equation (12) of [26]) is of the form,

(33) $$F\left(p\right)\;=\;C\;{\left[1-\frac{p^{2}}{2mE}\right]}^{[D(N-1)-2]/2},$$

where *m* is the mass of one particle, p is the momentum of one particle, *E* is the total energy of the system, *N* is the number pf particles in the system, and *D* is the dimensionality of space (in references [26,27] the authors refer to the spatial dimension as *f*, but we denote it by *D*, consistently with the rest of this review). The momentum distribution (33) is clearly a *q*-Gaussian with

(34) $$q\;=\;1+\frac{D(N-1)-2}{2}.$$

In their derivation of the single-particle momentum distribution (33), the authors use, as an intermediate step, the configuration distribution (30) discussed by Maxwell in [24]. As a matter of fact, the developments reported in [26] can, to some extent, be regarded as an interesting re-formulation and elaboration, from a modern point of view, of ideas that were already implicit in Maxwell’s seminal paper.

In [27], the authors provide a nice detailed discussion of the micro-canonical approach to a classical ideal gas in a uniform gravitational field confined to a *D*-dimensional vessel. As in reference [26], the system considered by the authors of [27] is assumed to have a finite number *N* particles and a total energy *E*. Its single-particle distribution, which is provided in Equation (10) of [27], is proportional to

(35) $${\left(1-\frac{p^{2}}{2mE}-\frac{mgz}{E}\right)}^{\left[(D/2)+1]N-[(D/2)+2\right]},$$

where *m* is the mass of a particle, p is the momentum of a particle, and z>0 denotes the height of particle measured from the bottom of the vessel. As a consequence of the above result, the single-particle distribution can then be expressed as

(36) $$F\left(z,p\right)\;=\;C\;{\left[1-\frac{\epsilon }{E}\right]}^{\left[(D/2)+1]N-[(D/2)+2\right]},$$

where C is an appropriate normalization constant and

(37) $$\epsilon \;=\;\frac{p^{2}}{2m}+mgz$$

is the single-particle energy. The distribution (36) has the $S_{q}$-canonical form. It is a *q*-exponential in the single-particle energy ϵ, with the *q* parameter given by

(38) $$q\;=\;1+{\left\{\left[\left(D/2\right)+1\right]N-\left[\left(D/2\right)+2\right]\right\}}^{-1}$$

The single-particle distributions studied in [26,27] fit into the general picture described in the previous section if one identifies system *A* with one of the system’s particles, and system *B* with the remaining N−1 particles.

## 5. The Many Facets of the $S_{q}$-Statistics-Micro-Canonical Connection

The connection between the micro-canonical ensemble and the *q*-exponentials and *q*-Gaussians for finite systems has been analyzed and discussed by several researchers, expanding this venue of inquiry into various interesting directions [28,29,30,31,32,33,34,35,36,37,38,39,40,41,42]. In this section, we shall review the main developments along these lines. We shall restrict our discussion to works centered on the $S_{q}$-canonical-micro-canonical relation per se, focusing on how the *q*-distributions arise from such a relation. Some of these works are presented in terms of finite or small heat bathes, but they contribute to our main concern here: how the *q*-distributions originate from micro-canonical scenarios. We shall not discuss works revolving around interpretative issues related to the finite-bath approach. Those constitute, no doubt, interesting and relevant efforts, but they are outside the scope of our present review.

The most powerful formulation of the micro-canonical approach to the $S_{q}$-thermostatistics for finite classical Hamiltonian systems with a continuous phase space is, arguably, the one advanced by Adib, Moreira, Andrade Jr, and Almeida (AMAA) in [29]. In terms of our discussion in Section 3, the main ideas in [29] can be interpreted as follows. Let us consider a finite classical system with a Hamiltonian that can be expressed as

(39) $$H\;=\;H_{A}\left(\omega_{A}\right)+\sum_{k=1}^{J}H_{k}\left(\omega_{k}\right),$$

where each of the terms $H_{A}$, $H_{1},\ldots ,H_{J}$, depends on a different subset of canonical variables. The term $H_{A}$ of the Hamiltonian depends on the subset $\omega_{A}$ of canonical variables, and each term $H_{k}\;\;\;\left(1\leq k\leq J\right)$ depends on the subset $\omega_{k}$. Each of the system’s canonical variables belongs to one and only one of the subsets $\left\{\omega_{A},\omega_{1},\ldots ,\omega_{J}\right\}$. Let us assume that each of the terms $H_{k}\;\;\;\left(1\leq k\leq J\right)$ depends on $n_{k}$ canonical variables (the set $\omega_{k}$ has $n_{k}$ components) and is a homogeneous function of degree $l_{k}$. That is, if

(40) $$\omega_{k}=\left(z_{1}^{\left(k\right)},\ldots ,z_{n_{k}}^{\left(k\right)}\right),\;\;\;\;\;\;\;\;k=1,\ldots ,J$$

is the set of $n_{k}$ canonical variables $z_{n_{k}}^{\left(k\right)}$ on which $H_{k}$ depends, one has,

(41) $$\begin{array}{ccc} H_{k}\left(\lambda \omega_{k}\right) & = & H_{k}\left(\lambda z_{1}^{\left(k\right)},\ldots ,\lambda z_{n_{k}}^{\left(k\right)}\right),\\ & = & \lambda^{l_{k}}\;\;H_{k}\left(\omega_{k}\right),\;\;\;\;\;\;\;\;\;\;\;\;\;\;\;\;\;\;\;\;\;\;\;\;k=1,\ldots ,J. \end{array}$$

Let $\omega_{A}=\left(z_{1}^{\left(A\right)},\ldots ,z_{n_{A}}^{\left(A\right)}\right)$ stand for the set of canonical variables on which the term $H_{A}$ depends. Then, the total number of canonical variables describing the complete system, is $n_{t}=n_{A}+\sum_{k=1}^{J}n_{k}$. The above requirements define a class of classical Hamiltonian systems that admits, as particular instances, concrete systems of practical relevance in Physics. For instance, the Hamiltonian corresponding to a system of *N* interacting classical point particles in *D* dimensions can be cast in the form (39), if one identifies the subset $\omega_{A}$ with the complete set of ND configuration coordinates, and the subset $\omega_{1}$ with the complete set of ND components of momenta. In this example, one has J=1, $n_{1}=ND$, and $l_{1}=2$. Other concrete examples will be mentioned later.

Under the above assumptions, the authors of [29] proved that, if the complete system is described by the micro-canonical distribution, then the marginal distribution corresponding to the variables $\omega_{A}$ has the $S_{q}$-canonical form. Let us take a closer look at this result from the view point of the general argument outlined in Section 3. Let us define $H_{B}$ as

(42) $$H_{B}\left(\omega_{1},\ldots ,\omega_{J}\right)\;=\;\sum_{k=1}^{J}H_{k}\left(\omega_{k}\right),$$

and consider the volume $\Phi_{B}\left(E\right)$ in the space characterized by the set of variables $\left(\omega_{1},\ldots ,\omega_{k}\right)$, for which $H_{B}\leq E$. We have

(43) $$\Phi_{B}\left(E\right)\;=\;\int_{H_{B}\left(\omega_{1},\ldots ,\omega_{J}\right)\leq E}\;\;\;d\omega_{1}\ldots d\omega_{J},$$

where

(44) $$d\omega_{k}\;=\;dz_{1}^{\left(k\right)}\cdots dz_{n_{k}}^{\left(k\right)},\;\;\;\;\;\;\;k=1,\ldots ,J.$$

Given a value *E* of the total energy and a reference value $E_{0}$, let us choose a set of dimensionless parameters $\lambda_{k}$ in such a way that

(45) $$\lambda_{k}\;=\;{\left(\frac{E}{E_{0}}\right)}^{1/l_{k}},\;\;\;\;\;\;\;k=1,\ldots ,J.$$

We then have, taking into account the homogeneity of the $H_{k}$s, that

(46) $$\frac{H_{k}\left(\lambda_{k}\omega_{k}\right)}{H_{k}\left(\omega_{k}\right)}=\lambda_{k}^{l_{k}}\;=\;\frac{E}{E_{0}},\;\;\;\;\;\;\;k=1,\ldots ,J,$$

which implies that

(47) $$\begin{array}{ccc} \Phi_{B}\left(E\right)\; & = & \;\int_{H_{B}\left(\omega_{1},\ldots ,\omega_{J}\right)\leq E}\;\;\;d\omega_{1}\ldots d\omega_{J}\\ & = & \;\left(\prod_{k=1}^{J}\lambda_{k}^{n_{k}}\right)\;\int_{H_{B}\left(\omega_{1}^{\prime },\ldots ,\omega_{J}^{\prime }\right)\leq E_{0}}\;\;\;d\omega_{1}^{\prime }\ldots d\omega_{J}^{\prime }\\ \; & = & \;\left(\prod_{k=1}^{J}\lambda_{k}^{n_{k}}\right)\;\Phi_{B}\left(E_{0}\right)\\ \; & = & \;{\left(\frac{E}{E_{0}}\right)}^{\sum_{k=1}^{J}\left(n_{k}/l_{k}\right)}\;\Phi_{B}\left(E_{0}\right). \end{array}$$

We then see that the volume $\Phi_{B}\left(E\right)$ grows as a power of *E*,

(48) $$\Phi_{B}\left(E\right)\;=\;\Phi_{B}\left(E_{0}\right)\;{\left(\frac{E}{E_{0}}\right)}^{\eta },$$

with

(49) $$\eta \;=\;\sum_{k=1}^{J}\frac{n_{k}}{l_{k}}.$$

One is then precisely within the scenario discussed in Section 3, from which it follows that the marginal probability density corresponding to the variables $\omega_{A}$ is of the $S_{q}$ canonical form

(50) $$F\left(\omega_{A}\right)\;=\;C{\left[1-\frac{H_{A}\left(\omega_{A}\right)}{E_{T}}\right]}^{\frac{1}{q-1}},$$

where $E_{T}$ is the total energy of the system, and

(51) $$q\;=\;\frac{\eta }{\eta -1}\;=\;1\;+\;{\left[\left(\sum_{k=1}^{J}\frac{n_{k}}{l_{k}}\right)-1\right]}^{-1}.$$

All the examples discussed in the previous section can be analyzed in terms of the AMAA formulation. In the systems discussed in [26,27], one has to identify the subsystem *A* with one particle of the system, and the subsystem *B* with the remaining N−1 particles. In the case discussed in [26], one has J=1, $n_{1}=D\left(N-1\right)$, and $l_{1}=2$. Then, (49) and (51) determine the values of η and *q*, and the latter coincides with (34). In the case discussed in [27], one has J=2, $n_{1}=D\left(N-1\right)$, $n_{2}=N-1$, $l_{1}=2$, and $l_{2}=1$. Then, from (49) and (51), one obtains the same value of *q* as (38).

Remarkably, AMAA performed numerical experiments on a system consisting of a chain of anharmonic oscillators, described by the Hamiltonian,

(52) $$H\;=\;\sum_{i=1}^{N}\frac{p_{i}^{2}}{2}\;+\;\sum_{i=1}^{N}\frac{q_{i}^{4}}{2}\;+\;\sum_{i=1}^{N}\frac{{\left(q_{i+1}-q_{i}\right)}^{4}}{4},$$

where $q_{i}$ and $p_{i}\;\;\;\;(i=1,\ldots ,N$ are the coordinates and momenta of the *N* particles in the system. The chain of anharmonic oscillators governed by the Hamiltonian (52) was inspired by the celebrated Fermi–Pasta–Ulam system. The Hamiltonian (52) has the form (39). For instance, if one identifies the set $\omega_{A}$ with the set of *N* momenta, and the set $\omega_{1}$ with the set of *N* coordinates (we then have J=1, $n_{1}=N$ and $l_{1}=4$), it follows that the marginal probability distribution corresponding to the momenta will be a *q*-exponential, provided that the system is described by the micro-canonical ensemble. The numerical experiments conducted by AMAA, based on the numerical integration of the canonical equations of motion, confirmed that the micro-canonical description is in this case appropriate, and that the probability distribution of the momenta is indeed *q*-exponential [29]. The numerical experiments reported by AMAA are of considerable relevance because they provide a concrete example of a highly nonlinear system, exhibiting complex dynamics, for which the micro-canonical path toward the $S_{q}$-canonical distributions is explicitly verified.

In [32], Hanel and Thurner provide a clever analysis of the general conditions under which micro-canonical scenarios in classical statistical mechanics naturally lead to power-law distributions of the *q*-exponential type. In [33], Naudts and Baeten point out that the configuration probability density of a classical gas always has the $S_{q}$-canonical form. Therefore, the authors establish the connection between this scenario and the $S_{q}$-thermo-statistics. The authors explore various aspects of this problem, and discuss the possible role of Renyi entropy (which, once optimized, shields the same probability distributions as Tsallis entropy, although parameterized in a different way).

In [34], Bagci and Oikonomou explore interesting aspects of the micro-canonical setting associated with a classical Hamiltonian system interacting with a finite heat bath. These authors focus on how different assumptions on the heat capacity of the bath lead to different versions of the $S_{q}$-canonical distribution. The authors reach the conclusions that a finite bath with positive heat capacity leads to *q*-exponentials with compact support (that is, with a cut-off), while a heat bath with negative heat capacity leads to *q*-exponentials with fat tails. Within the present review, we restrict our discussion to micro-canonical scenarios generating *q*-exponentials with a cut-off. We find, however, that Bagci and Oikonomuu’s analysis of situations with finite baths of negative heat capacity is intriguing and worthy of further consideration. In [35], Ramshaw re-visits the micro-canonical treatment of a system in contact with a finite heat bath. The author analyzes the concomitant deviations of the system’s probability distribution from the exponential Boltzmann–Gibbs one. The author’s analysis confirms that, under appropriate conditions, probability distributions of the $S_{q}$-canonical form are obtained. The author suggests that, although the $S_{q}$ entropy may play a direct role here, the presence of *q*-exponentials is not in itself enough to establish that that is the case. There may be something to the author’s point of view. This is an issue that certainly deserves further investigation. In [36], Biró, BarnafÏdi, and Ván apply the $S_{q}$-micro-canonical connection to the analysis of some aspects of the physics of the quark–gluon plasma and to the interpretation of experimental data on heavy ion collisions. An intriguing extension of these matters to complex values of the Tsallis parameter *q* is discussed by Wilk and Wlodarczyk in [37].

In [38], Lima and Deppman re-visit the micro-canonical approach to an ideal gas constituted by a finite number *N* of particles and provide a detailed analysis of its relation with the $S_{q}$-based thermostatistics. The authors investigate in detail how, as N→∞, the standard Boltzmann–Gibbs scenario is approached. In particular, they compute the two-particle correlation function and study how the amount of correlation decreases as one considers an increasing number of particles in the system. The micro-canonical ensemble for an ideal gas with a finite number of molecules is also considered by Shim in [39], where the $S_{q}$-canonical shape of the single-particle distribution is also investigated.

## 6. Conclusions

A growing number of generalizations of the concept of entropy are nowadays attracting the attention of scientists. Researchers are actively exploring diverse applications of these ideas, particularly in connection with entropic optimization methods. To a large extent, this broad field of inquiry got its initial inspiration in the $S_{q}$-thermostatistics advanced by Tsallis in 1988. The non-additive $S_{q}$ entropies still play a distinguished role within the zoo of generalized entropies. Among the different distributions optimizing non-standard entropies, those optimizing the $S_{q}$ measures are the ones that provide useful descriptions for the largest number of scenarios in physics and elsewhere. It is, therefore, imperative, in order to explain this state of affairs, to study in detail the particular features of the $S_{q}$-thermo-statistics that make it so special. The present review dealt with one of these distinguishing features: the remarkable connection between the $S_{q}$-thermostatistics and the micro-canonical ensemble. This connection is nowadays generally acknowledged as constituting the basis of an important mechanism generating $S_{q}$-optimizing distributions in physical systems. For instance, the significance of the fact that “research showed that finite ideal gas followed *q*-exponential distributions” [40], was recently highlighted by Deppman, a leading researcher in nuclear and particle physics, and in the application of Tsallis’ non-extensive statistics to these fields. Deppman and collaborators advanced an interesting theoretical framework, based on the concept of thermofractals, for the study of systems that exhibit a finite effective number of degrees of freedom, independently of the system’s size [41]. This scenario, which leads to a description similar to the micro-canonical one, generating *q*-distributions even for large systems, has been applied to problems in high-energy physics [41].

In spite of the considerable amount of research that has been devoted to investigate the micro-canonical approach to the $S_{q}$-canonical ensemble, we believe that the study of this aspect of $S_{q}$-thermostatistics is still in its infancy. There is still much work to be done, particularly in order to establish links between the micro-canonical setting and other facets of $S_{q}$-thermostatistics. In this regard, it would be interesting to explore Baranger’s conjecture that the micro-canonical approach may be relevant to explain the emergence of $S_{q}$-thermo-statistics within non-equilibrium scenarios [21]. A promising first step in this direction was taken by Megias, Lima, and Deppman in [42], where the authors consider transport phenomena on the light of the connection between the $S_{q}$-thermostatstical and the micro-canonical setting. Another issue that deserves further scrutiny is the connection of the entropy functional $S_{q}$ itself with the micro-canonical setting. Up to now, the main theoretical indication of a connection between the $S_{q}$-based thermostatistics and the micro-canonical scenario is based on the presence of the *q*-distributions, rather than on a more direct connection with the $S_{q}$ functional itself. The presence of *q*-distributions is generally construed, by a large part of the research community, as evidence for the $S_{q}$-based thermostatistics. This is the case not only with regards to the micro-canonical scenarios but also within more general contexts. In particular, most of the empirical evidence for the $S_{q}$-thermostatistics rests on the experimental observation of *q*-distributions [17,19]. This situation is not surprising since the distributions that describe a system are more amenable of experimental or observational investigation than an entropic functional. The fact that probability distributions optimizing a particular functional, the $S_{q}$ entropy, appear so frequently, both in theoretical models and in experimental settings, strongly suggests that the $S_{q}$ entropy is playing an important role. This is consistent with the general notion that variational principles provide the most fundamental description of physical systems or processes. It is significant, in this regard, that even researchers who doubt that the $S_{q}$-based optimization principle is the correct explanation for the presence of *q*-distributions, nevertheless entertain the idea that an alternative variational principle may be at work. For instance, Ramshaw recently observed that the non-exponential distributions describing subsystems in micro-canonical contexts, can be derived from an entropy-optimization prescription if one, instead of changing the entropic form, replaces the standard energy constraint by an adroitly chosen non-linear generalization [43]. Ramshaw’s proposal is intriguing, and certainly deserves further consideration. It nicely illustrates the belief of many theoreticians that the identification of an appropriate entropic variational principle leading to the non-exponential distributions observed on micro-canonical scenarios will contribute to achieving a deep understanding of these distributions. Among the proposals that have been advanced in this respect, the one based on the $S_{q}$-entropy has been, so far, the most actively investigated, as attested by the works reviewed here.

The $S_{q}$ canonical distributions, which exhibit the *q*-exponential (or, in particular cases, are *q*-Gaussian) form, arise naturally as marginal probability distributions describing parts of systems represented by the micro-canonical ensemble. In this regard, the *q*-distributions can be identified in some works dating from the very beginnings of statistical mechanics. They have also been appearing, along the years, in papers’ and textbooks’ discussions on the micro-canonical ensemble. The relevance of these ubiquitous distributions, however, was for a long time unrecognized. Until the development of the $S_{q}$ thermo-statistics, they lacked a name. The significance of their relationship with the micro-canonical ensemble, which links them to the origins and history of statistical mechanics, has been highlighted in recent times by the research efforts conducted by several scientists, who explored its interesting and manifold implications. Intriguing results along these lines continue to appear. What new developments the future will bring, we cannot tell. However, we can be sure of one thing: like Holly’s cat in *Breakfast at Tiffany’s* [44], these long-neglected distributions finally got a name, and the name is here to stay.

## Data Availability

Everything needed is contained in the paper.
